# Quality Characteristics and Essential Oil Properties of *Thymus capitatus*, *Mentha piperita*, and *Sideritis cypria* Dried under Different Conditions

**DOI:** 10.3390/plants13223150

**Published:** 2024-11-09

**Authors:** Panayiota Xylia, Antonios Chrysargyris, Ekaterina-Michaela Tomou, Christos Goumenos, Helen Skaltsa, Nikolaos Tzortzakis

**Affiliations:** 1 Department of Agricultural Sciences, Biotechnology and Food Science, Cyprus University of Technology, Limassol 3036, Cyprus; 2 Department of Pharmacognosy & Chemistry of Natural Products, School of Health Sciences, National and Kapodistrian University of Athens, 15771 Athens, Greece

**Keywords:** biological activities, drying process, energy consumption, mint, Cyprus ironwort, thyme

## Abstract

The drying of medicinal and aromatic plants (MAPs) is one of the main preservation methods for these products that can prolong their shelf life, if performed properly. The current study aimed to examine the effects of different drying conditions (sun, shade, and oven drying at 42 °C) on the quality characteristics of *Thymus capitatus*, *Mentha piperita*, and *Sideritis cypria*; their essential oil (EO) yield; and their biological properties (antioxidant and antibacterial activities). According to the results of the current study, oven drying resulted in faster moisture loss for all investigated species and slightly darker products. For *T. capitatus*, sun drying resulted in higher EO carvacrol content, whereas EOs obtained from shade and oven drying (at 42 °C) presented high total phenolic content and great antimicrobial activity. For *M. piperita*, shade drying resulted in a higher EO yield and higher *iso*-menthone content, whilst the EO obtained from oven-dried mint plants presented great antibacterial activity against the investigated foodborne pathogens. *S. cypria* plants dried in an air-ventilated oven produced an EO rich in β-caryophyllene and α-pinene, which also presented great antioxidant and antibacterial activity. The findings of the current study indicate that traditional drying methods, such as sun and shade, can result in good-quality dried MAPs that can yield EOs with significant biological activities, along with minimum energy consumption and lower carbon dioxide production (lower environmental carbon footprint), as opposed to oven drying. However, the drying-process duration could be a limitation at the industrial scale.

## 1. Introduction

Medicinal and aromatic plants (MAPs) with ethnopharmacological uses have been utilized as sources of natural remedies and healthcare for many years [1,2]. The commonly used herbal preparations are decoctions and/or infusions; they are also launched in the market in different pharmaceutical formulations [3]. Novel and contemporary medicines based on MAPs and natural compounds are popular since MAPs are considered excellent sources of phytochemicals with curing and preventive properties [4,5]. In addition, there is increased interest in the use of the essential oils (EOs) from these products as alternative, natural antimicrobial agents. As the outbreaks of foodborne illnesses increase, the search for synthetic antibiotic alternatives also increases. The most frequently reported cases related to foodborne illnesses include infections with foodborne pathogens such as *Salmonella* spp., *Escherichia coli*, *Listeria monocytogenes*, *Camplylobacter perfigens*, *Shigella* spp., and *Staphylococcus aureus*, among others [6,7,8]. Some of these pathogens could result in high mortality rates, along with the increased antibiotic resistance; thus, it is crucial that new agents are employed for their control and elimination [9].

Fresh and dry MAPs are used in everyday life, as well as in the medical, cosmetic, industrial, and culinary fields. Mint (*Mentha piperita* L.) is considered to be among the most popular and widely consumed and used MAPs [10,11]. Another species of great interest is *Thymus capitatus* (L.) Hoffmanns & Link (*Thymbra capitata* L., also known as throumpínn or throumpi, ágrio thymári, cone head thyme, and Persian hyssop), which is used in traditional treatments for numerous illnesses, including flu, cough, diabetes, dermatitis, indigestion, respiratory disorders (including asthma), rheumatic, and diarrhea among others [12,13,14]. This herb was found to present great antioxidant and antimicrobial activities [12,15]. In addition, *Sideritis cypria* Post. infusions are locally used in Cyprus against stomach disorders, headache, and the common cold, as they contain antioxidant and antimicrobial properties [16,17,18].

Drying is one of the most known and widely used methods applied in the food industry for the preservation of fruits, vegetables, and herbs. This process aims to remove the moisture content of the product and, at the same time, decrease its volume and weight, as well as the costs of packaging, storage, and transportation [19]. By lowering the product’s moisture content (up to 5–13%), the quality of the product is ensured due to minimizing the growth of spoilage microorganisms and inhibiting the chemical reactions which evidently will result to quality losses and food waste [20,21]. The process of drying used to be a natural and simple procedure since ancient times by exploiting solar energy. Nowadays, this procedure is more sophisticated, using different equipment (oven, freeze dryer, microwave, etc.), and researchers are investigating different drying parameters for the optimization of the drying conditions for each investigated MAP [19]. However, if the drying process takes place in non-optimal conditions (i.e., too-high temperature) the quality of the end product can undergo to undesirable changes, such as leaf browning/yellowing, development of off-flavors (aroma, texture, and taste), and degradation of phytochemicals [10,19].

Traditionally, the drying of herbs took place by placing the plant material under direct sunlight in a thin layer. Previous studies highlighted that some MAPs are not suitable for sun drying, since observations were made regarding the loss of their distinct aroma and the degradation of their color [20]. Another traditional drying method used is shade drying, where MAPs are placed (in a thin layer) away from direct sunlight (i.e., shade), in a well-aerated room with low relative humidity. This method is usually applied for MAPs that present sensitivity to light (avoiding the oxidation of phytochemicals and EO compounds) [22]. Nowadays, oven drying (convective drying) is widely applied in different industries. During this method, herbs are exposed to hot air at a set temperature in air-ventilated ovens. Even though this method is faster and the conditions can easily be monitored without being affected by the weather conditions (as opposed to sun drying), a set temperature is applied for all MAPs, without taking into account the different requirements of each species [21]. On the other hand, oven drying relies on electricity and fuels, making this process not environmentally friendly, as opposed to natural drying processes, like sun and shade drying. Since consumers prefer and search for products produced under sustainable conditions and with a low-environmental-footprint process, there is a need for research into the optimum drying conditions for specific MAPs.

The current work aimed to investigate the effects of different drying conditions (i.e., sun, shade, and oven) on *T. capitatus*, *M. piperita*, and *S. cypria* quality attributes and their EO production (i.e., yield, composition, and biological properties) in order to select the optimum conditions for each MAP. In addition, the energy consumption and carbon footprint of the investigated drying conditions were determined for the selection of more environmentally friendly drying procedures.

## 2. Results

### 2.1. Effects on Moisture Content, Drying Time, Energy Consumption, and CO_2_ Production

**Figure 1:**
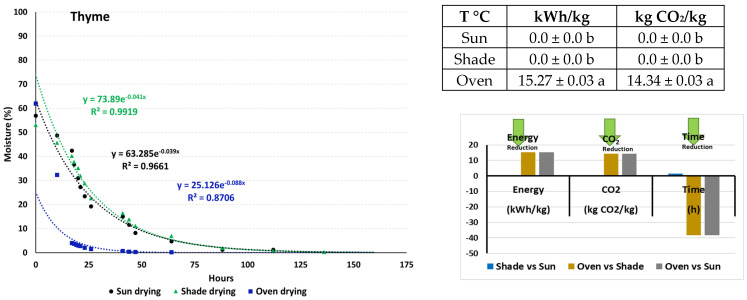

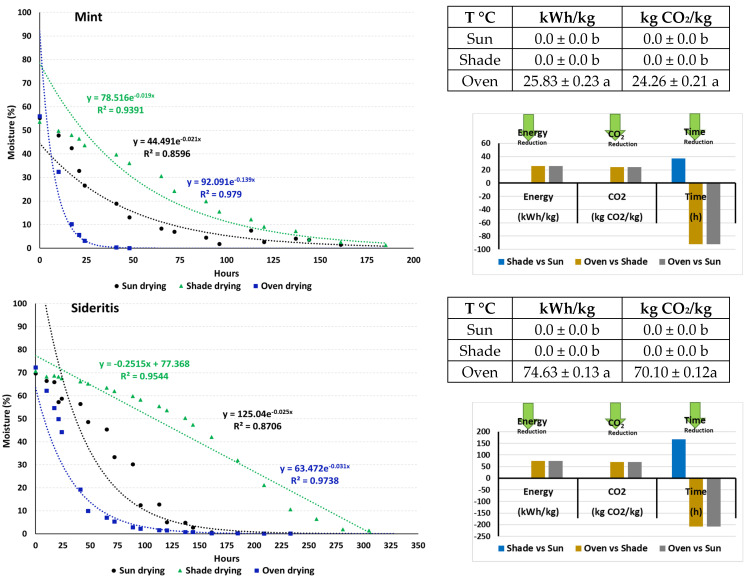
Effects of drying conditions (sun, shade, and oven) on *T. capitatus*, *M. piperita*, and *S. cypria* moisture content, drying time, energy consumption, and CO_2_ production. Values are expressed as means ± standard (n = 3). Values in columns for energy consumption and CO_2_ production that are followed by different letters are significantly different (*p* < 0.05).

The following equations describe the moisture decrease in *T. capitatus*: y = 63.285 e^−0.039x^ (R^2^ = 0.9661) at sun drying, y = 73.89 e^−0.041x^ (R^2^ = 0.9919) at shade drying, and y = 25.126 e^−0.088x^ (R^2^ = 0.8706) at oven drying (Figure 1). Considering that the initial moisture content of *Thymus* was 55%, and using the previously stated equations, the time needed for moisture reduction from 55% to 10% is T_sun_ = 47.3 h, T_shade_ = 48.8 h, and T_oven_ = 10.5 h. Thyme plants dried in an oven consumed energy, i.e., 15.27 kWh/kg; produced CO_2_, i.e., 14.34 kg CO_2_/kg; and shortened the drying time up to 38.3 h compared to the plants dried under sun or shade. The application of shade versus sun conditions delayed the drying time by up to 1.49 h.

For *M. piperita*, the following equations describe the moisture decrease: y = 44.491 e^−0.021x^ (R^2^ = 0.8596) at sun drying, y = 78.516 e^−0.019x^ (R^2^ = 0.9391) at shade drying, and y = 92.091 e^−0.139x^ (R^2^ = 0.979) at oven drying (Figure 1). Taking into consideration that the initial moisture content of mint was 65%, and using the previously stated equations, the time needed for moisture reduction from 65% to 10% is T_sun_ = 71.1 h, T_shade_ = 108.5 h, and T_oven_ = 16.0 h. Peppermint plants, after drying in an oven, consumed energy, i.e., 25.83 kWh/kg; produced CO_2_, i.e., 24.26 kg CO_2_/kg; and shortened the drying time up to 92.49 h compared to the plants dried under sun or shade. The application of shade versus sun conditions delayed the drying time by up to 37.38 h.

In *S. cypria*, the moisture decrease is described by the following equations: y = 125.04 e^−0.025x^ (R^2^ = 0.8706) at sun drying, y = −0.2515x + 77.368 (R^2^ = 0.9544) at shade drying, and y = 63.472 e^−0.031x^ (R^2^ = 0.9738) at oven drying (Figure 1). Considering that the initial moisture content of *S. cypria* was 70%, and using the previously stated equations, the time needs for moisture reduction from 70% to 10% are T_sun_ = 101.0 h, T_shade_ = 267.9 h, and T_oven_ = 59.6 h. Cyprus ironwort plants, after drying in an oven, consumed energy, i.e., 74.63 kWh/kg; produced CO_2_, i.e., 70.10 kg CO_2_/kg; and shortened the drying time by up to 208.25 h compared to the plants dried under sun or shade. The application of shade versus sun conditions delayed the drying time by up to 166.82 h.

### 2.2. Impact on Chlorophyll Content and Leaf Color

A higher chlorophyll content (i.e., Chl a, Chl b, and Total Chl) was observed with oven drying of *T. capitatus*, as opposed to sun drying (Table 1). However, *Thymus*’s color parameters (hue, chroma value, color index, and browning index) were not significantly affected by the investigated drying conditions. Regarding the chlorophyll content of *M. piperita*, no significant differences were reported with the investigated drying conditions (sun, shade, and oven drying), whilst mint plants dried under shade and in an oven showed higher chroma values and browning index values compared to sun drying, suggesting a dark-colored product (Table 1). Sun drying of *S. cypria* resulted in decreased chlorophyll content (i.e., Chl a, Chl b, and Total Chl) compared to oven drying at 42 °C (Table 1). A higher color index was found with the shade drying of sideritis plants compared to the other drying conditions, whereas oven drying showed a higher chroma value compared to sun and shade drying (Table 1). The hue angle was higher with sun and oven drying compared to shade drying. An increased browning index was observed during the oven drying of sideritis, indicating a darker product compared to the other drying conditions.

### 2.3. Impact on EO Yield and Composition

An analysis of the EOs of *T. capitatus* plants dried under different conditions is presented in Table 2. Twenty-seven compounds (>0.05%) were identified, a majority of which belong to the group of oxygenated monoterpenes (70.27–75.46%), followed by monoterpenes hydrocarbons (22.34–27.72%). The two dominant compounds of the EO of thyme plants were carvacrol (34.56–38.86%) and thymol (27.47–29.86%), and components such as p-cymene, γ-terpinene, and borneol followed. The EO extracted from plants dried under the sun and in an air oven revealed significantly higher amounts of carvacrol and borneol compared to the plants dried under shade; the latter appears to be richer in γ-terpinene. Compounds such as thymol, α-pinene, and β-myrcene remained unaffected by the different drying conditions, while p-cymene appeared increased when plants were dried under the sun or in the shade. The EO yield was not affected by the different drying conditions tested and ranged between 1.75 and 1.92%.

The effect of the different drying conditions on the yield and quality of the EO extracted from *M. piperita* plants is illustrated in Table 3. The EO analysis revealed the presence of thirty individual compounds, which represented 99.62–99.82% of the total oil. Oxygenated monoterpenes made up the most abundant group of compounds (93.31–93.70%), followed by monoterpenes hydrocarbons (4.45–4.82%). Menthol is the major compound of *M. piperita* EO, and it ranged from 41.93 to 44.45%. Other compounds identified are (in decreasing order) *iso*-menthone (16.25–18.69%), menthyl acetate (14.00–16.95%), eucalyptol (7.46–8.20%), menthone (2.80–3.78%), and D-limonene (2.12–2.34%). The rest of the identified components were found to represent amounts lower than 2% of the total volatile composition. The different drying conditions had a significant effect on the major compounds of the EO of mint plants. Menthol appeared to increase when plants were dried in an oven. Compared to the sun-dried plants, *iso*-menthone reached the highest amount (18.69%) in plants under shade drying, while sun dried plants had the highest level of menthyl acetate (16.95%). Plants dried under shade had the highest EO yield (1.59%) compared to the sun-dried plants (1.38%), but there was no significant difference from the EO yield of the oven-dried plants (1.48%).

The analysis of the EO of *S. cypria* plants dried under the sun, in the shade, or in an air oven revealed the presence of twenty-six compounds (>0.05%), while the majority of them belonged to monoterpenes hydrocarbons (69.67–74.66%) (Table 4). Both sesquiterpene hydrocarbons and oxygenated ones follow, ranging from 10.05 to 13.68% and from 9.95 to 12.48%, respectively. The group with the lower representation in the EO profile was the oxygenated monoterpenes (3.31–5.12%). The major compounds were β-phellandrene (33.81–37.72%), β-caryophyllene (9.74–13.25%), α-pinene (11.21–11.84%), sabinene (10.17–11.42%), caryophyllene oxide (8.75–11.01%), and β-pinene (6.10–8.27%). Compared to the other tested MAP species, the effect of drying conditions on the quality of the EO was more profound in the case of *S. cypria*. All the major compounds were significantly affected by the drying conditions, as presented in Table 4. The EOs obtained from the sun-dried plants presented a high amount of sabinene (11.25%) and caryophyllene oxide (11.01%), the EOs isolated from shade-dried plants showed increased sabinene (11.42%) and β-phellandrene (37.72%), and the EOs from air-oven dried plants were richer in β-caryophyllene (13.25%), and α- and β-pinene (11.84% and 8.27%, respectively). The plants’ low EO yield (compared to the other investigated MAP species) was not affected by the different drying treatments, and it was averaged at 0.23%.

### 2.4. Impact on EOs Biological Activities

#### 2.4.1. Antioxidant Activity

*T. capitatus* EOs were found to be rich in phenolic compounds (Table 5), especially the EOs derived from plants dried under shade or in an oven (65.18 and 63.73 mg GAE/g of EO, respectively). An EO with higher total antioxidant capacity and antioxidant activity (ABTS and DPPH) was obtained from shade-dried plants, and in some cases, it was almost 300 times higher than the activity of the reference compounds (BHT and ascorbic acid). As presented in Table 5, the total phenolic content and the total antioxidant capacity of *M. piperita* EOs were not affected by the different drying conditions, while the DPPH assay revealed significantly lower activity of all mint EOs compared to the antioxidant reference compound. The ABTS assay showed that the EOs obtained from oven drying mint plants presented antioxidant activity higher than those of shade- or sun-dried plants, while the activity was similar to the reference standard (19.39 and 19.62 μg/mL, respectively).

The effect of the drying conditions on the EO from *S. cypria* plants is presented in Table 5. An EO with the highest total phenolic content (4.63 mg GAE/g EO) was obtained from shade-dried plants; while sun- and shade-dried plants had EOs with a significantly high total antioxidant capacity compared to oven-dried plants. The DPPH assay showed that all the tested EOs had significantly lower activity than BHT (reference antioxidant compound), and EOs extracted from sun- and oven-dried plants presented the lowest antioxidant activity (higher IC50). The tested ABTS assay revealed the high potential of the EOs from sun dried plants, while all EOs had lower IC50 values (higher potential) than the ascorbic acid (used as reference compound).

#### 2.4.2. Antibacterial Activity

The effects of the drying conditions on the antimicrobial activity of *T. capitatus* EOs are shown in Table 6. *T. capitatus* EOs extracted from plants dried under shade or in an air oven presented lower IC50 values (i.e., greater antimicrobial activity) against *S. enterica* and *S. aureus*; however, this activity was lower than that of streptomycin (reference antibiotic). In addition, great antimicrobial activity against *L. monocytogenes* was also observed with the EOs obtained from the oven-dried plants compared to other drying conditions. Regarding *M. piperita* EOs, drying in an air oven resulted in greater antibacterial activity against *E. coli* and *S. enterica* (lower IC50 and MIC values, respectively) (Table 6). However, this effect was lower than the activity of the reference antibiotic (streptomycin). In addition, EOs isolated from shade- and oven-dried plants showed greater antibacterial activity (i.e., lower IC50 values) against *S. aureus* compared to the EOs from the sun-dried plants.

Table 6 presents the effects of the drying conditions on the antibacterial activity of *S. cypria* EOs. EOs obtained from oven-dried *S. cypria* plants presented greater antimicrobial activity (lower MIC and IC50 values) against *E. coli* and *S. enterica* compared to the EOs resulting from sun and shade drying but lower than streptomycin. No significant differences among the EOs obtained from plants dried under the investigated drying conditions were reported for *S. aureus* and *L. monocytogenes*. Among the tested bacteria, *S. aureus* (Gram-positive spherical-shaped bacteria) was found to be the most susceptible to the investigated EOs, followed by *L. monocytogenes* (Gram-positive rod-shaped bacteria), compared to the other two Gram-negative, rod-shaped bacteria (*E. coli* and *S. enterica*).

## 3. Discussion

Drying is an ancient, common, and physical preservation procedure for MAPs which is used for the direct preparation of dried herbs, as well as plant material used for further processing in the pharmaceutical, cosmetic, and food industries, among others. This process used to be a natural and simple technique to remove the moisture of herbs using solar energy. However, nowadays, this process has become more complex by using different equipment (oven, freeze dryer, microwave, etc.) and investigating different drying parameters for the optimization of the drying conditions for each investigated MAP [19,21]. It is worth mentioning that if the drying takes place in non-optimal conditions (i.e., too high temperature and high relative humidity), the quality of the end product will be lower, since the plant tissue can undergo undesirable changes, such as leaf browning/yellowing, development of off-flavors (aroma, texture, and taste), and degradation of phytochemicals [10,19]. In addition, the optimum drying method and related cost of the procedure are different for each MAP species investigated, and the conditions applied in each case must be recommended after proper examination.

During the drying process, moisture (i.e., water) is removed from the plant tissue (surface and inside), resulting in solid-dried products. Two types of moisture exist in a fresh plant tissue: (i) bound moisture (the water retained in microstructures) and (ii) unbound moisture (the excess of the bounded water) [19,23]. The moisture content of the fresh plant material is essential for the duration of the drying process and the achievement of the required moisture level of the end-dried product. By lowering the moisture content, the water activity (a_w_) of the product also decreases, eliminating the possibility of microbial growth (spoilage and pathogenic microorganisms) [24,25]. In addition, it has been previously reported that drying (if not performed correctly) might result in the deterioration of sensory attributes (i.e., aroma and taste) and the loss of physicochemical and nutritive components [19].

The conditions of the drying process greatly affect the duration of this procedure until the required moisture level is reached. Among the main factors that influence the duration of drying is the temperature and the relative humidity [26]. Other factors that influence the duration of the drying process include the volume, the plant organ, and the thickness of the organ, as well as its initial moisture content. It is worth mentioning that a longer drying time has been related to greater quality degradation, as well as greater energy consumption and a higher carbon footprint. In the current study, although the sun and shade drying took longer to complete compared to oven drying, these drying methods do not use any electricity and fuels (unlike for oven drying). The faster method of drying for all investigated MAP species was oven drying, followed by sun drying. Thus, choosing the correct drying method and conditions is essential for determining the environmental and economic aspects and their effects of the characteristics of the end product, as well as the environmental carbon footprint of this process [21,27].

As happens with fresh herbs, the color of the dried product is of great essence and influences the purchasing decisions of consumers. The loss of the green color of a dried product is assumed to be related to the loss and/or degradation of leaf chlorophylls and the oxidation of phenolic compounds, leading to the development of brown-colored compounds (i.e., o-quinones) [24]. In the current study, the chlorophyll content of *T. capitatus* and *S. cypria* was found to decrease during sun drying, as opposed to oven drying, whilst *M. piperita* chlorophyll content was not affected by the investigated drying conditions. These observations could be attributed to the degradation of chlorophylls due to exposure to the direct sunlight. However, one must not ignore the fact that chlorophylls a and b can transform into their epimers (chlorophyll a′ and b′) and/or other derivatives, which present almost the exact spectrum as their non-prime forms, which could be perceived as the preservation of chlorophyll content [28]. A quick dehydration of a plant tissue during the drying process could possibly reduce the Maillard reaction, resulting in a less brown color of the product.

One of the main products that can be derived from MAPs is their EOs. The EOs are natural complexes of volatile. These products are well known and used for centuries with a variety of applications, including medicinal–therapeutic and ritual purposes [29]. During the drying process and the removal of water from the plant tissue, a possible movement of volatile compounds might occur with a negative effect on the aroma, taste of the dried material, and the EO’s properties [30]. Several studies have investigated the effect of drying methods on the EO volatile composition of MAPs, indicating that the chemical content could be highly influenced [31]. For instance, the increase and decrease in volatile concentration and the grouped components or the formation of new chemical compounds could be observed [31]. In the present study, the EO yields from *T. capitatus* and *S. cypria* were not significantly affected by the investigated drying conditions (i.e., sun, shade, and oven drying), whereas a higher EO yield was found on shade-dried *M. piperita* plants. This phenomenon might be due to the fact that the investigated MAPs are part of the Lamiaceae family, and the members of this family are known to store their EOs on their leaf surfaces, making the loss of their volatile compounds easier [20,32]. From the current study, it seems that various MAP EOs are affected differently by the drying conditions due to their different leaf surface structures.

As changes in the EO yield could happen due to the applied drying conditions, changes in their major and minor components also could occur, influencing their biological activities. In the present study, in various ways, different drying conditions influenced the antioxidant activity of the obtained EOs from assorted MAPs. Indeed, EOs isolated from *T. capitatus* and *S. cypria* oven-dried plants presented higher total phenolic content and antioxidant capacity, whilst *M. piperita* EOs’ phenolic content and total antioxidant capacity were not affected by the drying conditions. The increase in phenolic content and antioxidants in EOs is of high importance since it signals high activity and benefits the human health. By increasing the major components of the investigated EOs (i.e., carvacrol, thymol, menthol, β-phellandene, and α and β-pinene) the possibility of preventing and treating illnesses related to oxidative stress and inflammation automatically increases too [33,34].

The antibacterial activity of EOs has been known for decades, and the use of EOs as alternative antibiotic agents is gaining great interest by the pharmaceutical and research community in an attempt to fight drug-resistant strains. From the findings of the current study, it was obvious that *L. monocytogenes* and *S. aureus* (i.e., Gram-positive bacteria) were found to be more susceptible to the tested EOs compared to *S. enterica* and *E. coli* (i.e., Gram-negative bacteria). In fact, Gram-positive bacteria have previously been reported as more susceptible to EOs’ activity compared to Gram-negative bacteria [35,36], as the bacterial membrane of the Gram-negative bacteria consists of an outer lipid membrane that seems to inhibit the interference of EOs’ lipophilic compounds with the inside bacterial components [34,37]. The main mechanisms of the EOs against bacteria include cell-wall damage, DNA damage, protein denaturation, enzyme inactivation, and anti-quorum sensing activity, among others [15,35,37]. In addition, *T. capitatus* and *M. piperita* EOs presented greater antibacterial activity compared to *S. cypria*, and this observation might be related to the general composition of each EO. As previously stated, EOs that are rich in monoterpenes and sesquiterpenes present greater antimicrobial activity as opposed to EOs that are rich in hydrocarbons [29]. Indeed, the previous statement confirms the observations of the current study, where *T. capitatus* and *M. piperita* EOs were found to be rich in oxygenated monoterpenes, while *S. cypria* EOs were rich in monoterpene hydrocarbons. The hydrophobic nature of terpenes enables them to interfere with the phospholipids of the cell membrane, altering the permeability of the bacterial cell wall’s membrane and disrupting its integrity, eventually resulting in the death of the bacterial cells [29,38]. The findings from the current study indicate that the increase in the major compounds of EOs also resulted in greater antibacterial activity.

## 4. Materials and Methods

### 4.1. Plant Material

Thyme (*Thymus capitatus*), mint (*Mentha piperita*), and sideritis (*Sideritis cypria*) plants were grown for eight months in a commercial organic farm located in Limassol, Cyprus (34°44′16.53″ N and 32°44′40.86″ E, 427 m above sea level). The area of the farmland (approximately 1000 m^2^) where the plants were cultivated consisted of a clay loam-texture soil that had 4.61% organic matter, available CaCO_3_ at 60.48%, pH 7.66, EC at 1.10 mS/cm, N at 2.46 g/kg, P at 0.06 g/kg, and K at 0.36 g/kg. Shortly after harvesting (September 2023), the plant material was prepared into small, homogenous bundles and transferred, avoiding moisture loss, leaf wilting, and/or degradation. In total, 45 bundles were prepared (5 replications/bundles per condition).

### 4.2. Drying Conditions

Each bundle’s weight was recorded prior to its placement on an aluminum tray (dimensions: 32 × 25 × 4.5 cm) (one bundle per tray, avoiding the compression of the plant tissue). Three sets of trays were prepared, and each set was exposed to a different drying condition (i.e., sun, shade, and oven). For the sun drying, the trays were placed under direct sunlight at the experimental farmland/greenhouse of Cyprus University of Technology (approximately 30 cm from the ground). For shade drying, the trays were placed in a dark room at the greenhouse that was aerated through open side and roof windows. For the oven drying, the trays were placed in an air-ventilated oven (SANYO convection oven, MOV-212F, SANYO Electric Co., Ltd., Osaka, Japan), at 42 °C, at the laboratory. The weight of each bundle was periodically measured (in the beginning of the drying process, every 6 h; and afterwards, every 12 and 24 h, when the weight loss rate was slower) until constant weight. After the recording of each weight, the plant material was manually mixed and turned. For the oven drying, the oven operated at 42 °C for at least 30 min in order to reach a steady state prior to placing the plant material inside.

### 4.3. Measurements

#### 4.3.1. Moisture Content, Drying Time, Energy Consumption, and CO_2_ Production

The moisture content, as a percentage (%), was estimated based on the weight loss at every recording time point. Graphs of the moisture content (%) in relation to the drying time were prepared by plotting the values recorded at every time point. From this, curves and equations were generated and fitted to the experimental points, maximizing the R^2^ (determination coefficient) in order to estimate the drying time required (in hours) to reach a definite moisture content (i.e., 10%).

For oven drying, the energy consumption was measured with the use of an energy power meter (Energenie ENER007, Bicester, UK), which was connected to the drying process. The energy consumed and the produced carbon dioxide (CO_2_) emissions due to the oven operation were determined. The accumulated energy and the carbon footprint were also estimated according to the equations reported by Ibrahim et al. [27].

#### 4.3.2. Chlorophyll Content and Leaf Color

The determination of the chlorophyll content (chlorophyll a, chlorophyll b, and total chlorophyll) was performed according to the method used by Nagata and Yamashita [39], and the results were reported as mg of chlorophyll per g of dried weight (mg/g Dw). The color of the dried MAPs was evaluated with the determination of the following parameters: hue (dominant color), chroma value (intensity of a color), color index, and browning index [36,40]. A colorimeter (Chroma meter CR400 Konica Minolta, Tokyo, Japan) was used to measure the L* (brightness/lightness), a* (greenness/redness), and b* (blueness/yellowness) color coordinates of the CIELAB uniform color space.

#### 4.3.3. Essential-Oil Extraction and Composition

A Clevenger apparatus was used for the hydrodistillation of the dried plant tissues and the extraction of their EOs (duration: 3 h). The yield of the EOs was estimated as μL of EO per 100 g of dry weight of the plant material (*v*/*w* on dry weight basis) and presented as a percentage (%). The collected EOs were stored at −20 °C in amber glass vials, until analysis. The analysis of the EOs constituents was carried out by analyzing the EOs with a Shimadzu GC2010 gas chromatograph-interfaced Shimadzu GC/MS QP2010 plus mass spectrometer (Shimadzu Corporation, Kyoto, Japan), according to the conditions previously reported by Chrysargyris et al. [41]. Retention indices were calculated for all the compounds through the use of a homologous series of C_8_–C_20_ *n*-alkanes on the same chromatographic conditions, according to the Van den Dool and Kratz approach [42]. The identification of the chemical components was based on a comparison of both the relative retention times and mass spectra with those reported in the NIST08 and literature data, as described by Adams [43], as well as with a series of authentic compound standards available in our laboratory.

#### 4.3.4. Estimation of ΕOs Total Phenolic Content and Antioxidant Activity

The total phenolic content of the obtained EOs was determined with the Folin–Ciocâlteu colorimetric assay, using the homonymous reagent [44]. The reaction’s optical density (OD) was read at 755 nm, and the results were expressed as mg of gallic acid equivalents per gram of EO (mg GAE/g oil). Three different assays were employed for the investigation of the EOs antioxidant activity, namely (i) total antioxidant capacity (TAC), (ii) 2,2-diphenyl-1-picrylhydrazyl (DPPH) radical scavenging activity, and (iii) 2,2′-azinobis-(ethylbenzothiazoline-6-sulfonic acid) (ABTS) radical scavenging activity. The total antioxidant capacity of the EOs was determined according to Kumar et al. [45], by measuring their ability to reduce Mo (VI) into Mo (V). Butylated hydroxytoluene (BHT) (Sigma-Aldrich, Darmstadt, Germany) was used as the standard compound, and results were expressed as equivalents of ascorbic acid per g of EO (mg AAE/g oil). The ability of the EOs to scavenge the DPPH free radical was measured by the procedure described by Oke et al. [46], but slightly modified. The OD of the reaction was measured at 517 nm, and the scavenging activity was expressed as the inhibition percentage (%), whereas the antiradical activity was expressed as the concentration of the sample/reference that results in 50% of inhibition (IC50). For this assay, BHT was used as a positive reference. The EOs’ scavenging activity against the ABTS radical was determined according to Wang et al. [47], with a few modifications. The reaction’s absorbance was measured at 734 nm, and the antioxidant activity was expressed as IC50, using the ascorbic acid as a reference compound.

#### 4.3.5. Determination of Essential Oils Antibacterial Activity

To test the antibacterial activity of the investigated EOs, four bacteria were selected as follows: *Escherichia coli* (ATCC 25922), *Salmonella enterica* subsp. *enterica* (ATCC 51741), *Staphylococcus aureus* (ATCC 11632), and *Listeria monocytogenes* (ATCC 19111). The microdilution method was used for the determination of the minimum inhibitory concentration (MIC) and the inhibitory concentration that can reduce the bacterial population by 50% (IC50), as previously described [36,48]. Briefly, 45 μL of EO dilution was added to 50 μL of brain heart infusion broth (BHI, Biokar, Beauvais Cedex, France) and 5 μL of overnight bacterial culture (10^6^ cfu/mL) in a sterile 96-well plate. Afterwards, the OD was measured at 630 nm in a microplate reader (ELx808 BioTek Instrument Inc., Highland Park, VT, USA) every 30 min during a 22 h incubation at 37 °C. For each EO, eight two-fold serial dilutions were prepared from a stock solution (20 mg/mL) prepared in dimethyl sulfoxide (DMSO; Merck, Darmstadt, Germany). In addition, the activity of the EOs was compared to the activity of streptomycin (used as a reference antibiotic substance). To secure that sterile conditions were applied during the preparation of each plate, positive and negative controls (PC and NC, respectively) were prepared every time as follows: PC—bacterial culture and BHI broth; NC1—EO dilution and BHI broth; and NC2—BHI broth. In addition, each bacterium was grown under different DMSO dilutions, verifying that the initial DMSO concentration (25%) used for the preparation of the EOs stock solutions did not affect the bacterial growth. Results were expressed as μg/mL.

### 4.4. Data and Statistical Analysis

The present study followed a Completely Randomized Design (CRD), where each treatment consisted of five biological replications (EO composition and biological activities consisted of three replications). One-way analysis of variance (ANOVA) was used for the statistical analysis of the obtained data in IBM SPSS version 25.0. Furthermore, Duncan’s multiple range test was applied to compare the means among the investigated treatments (significance level: *p* = 0.05).

## 5. Conclusions

The present study investigated the effects of different drying conditions (sun, shade, and oven drying at 42 °C) on the quality characteristics of *T. capitatus*, *M. piperita*, and *S. cypria* and their EOs in an attempt to propose the optimum drying scenario for each species investigated. For *T. capitatus*, sun drying was found to produce an EO rich in carvacrol that also presented great antioxidant activity, whilst shade and oven drying presented great antibacterial activity. Shade-dried *M. piperita* plants presented a higher EO yield and higher *iso*-menthone content, whilst mint EO obtained from oven drying presented great antibacterial activity against the investigated foodborne pathogens. For *S. cypria*, drying at 42 °C in an air-ventilated oven resulted in an EO rich in β-caryophyllene and α-pinene with great antioxidant and antibacterial activity. The findings of the current study suggest that different MAPs are affected differently by the different drying conditions. In addition, sun- and shade-drying conditions might present similar results (high-quality dry product and EOs with high biological activities) to oven drying (42 °C), contributing to a lower environmental carbon footprint.

## Figures and Tables

**Table 1:** Effects of drying conditions (sun, shade and oven) on *T. capitatus*, *M. piperita*, and *S. cypria* color and chlorophyll content (chlorophyll a, Chl a; chlorophyll b, Chl b; and total chlorophyll, Total Chl).

Plant		Sun Drying	Shade Drying	Oven Drying
*T. capitatus*	Chl a (mg/g)	0.38 ± 0.00 b	0.39 ± 0.01 ab	0.41 ± 0.01 a
	Chl b (mg/g)	0.61 ± 0.02 b	0.65 ± 0.01 ab	0.70 ± 0.02 a
	Total Chl (mg/g)	1.00 ± 0.02 b	1.04 ± 0.01 ab	1.11 ± 0.03 a
*M. piperita*	Chl a (mg/g)	0.38 ± 0.02	0.35 ± 0.01	0.37 ± 0.00
	Chl b(mg/g)	0.53 ± 0.04	0.45 ± 0.03	0.52 ± 0.03
	Total Chl (mg/g)	0.91 ± 0.06	0.81 ± 0.04	0.89 ± 0.03
*S. cypria*	Chl a (mg/g)	0.36 ± 0.01	0.37 ± 0.01	0.36 ± 0.01
	Chl b(mg/g)	0.45 ± 0.04 b	0.53 ± 0.01 ab	0.58 ± 0.02 a
	Total Chl (mg/g)	0.82 ± 0.04 b	0.90 ± 0.01 ab	0.94 ± 0.02 a
		**Sun Drying**	**Shade Drying**	**Oven Drying**
*T. capitatus*	Hue angle (°)	101.62 ± 1.95	104.63 ± 0.35	104.47 ± 2.25
	Chroma value	13.12 ± 0.47	14.60 ± 0.47	13.13 ± 0.55
	Color index	−4.48 ± 0.76	−5.82 ± 0.21	−5.81 ± 0.92
	Browning index	27.46 ± 1.66	30.28 ± 1.31	26.88 ± 1.02
*M. piperita*	Hue angle (°)	110.10 ± 1.48	109.67 ± 1.81	113.49 ± 1.12
	Chroma value	13.56 ± 0.46 b	17.39 ± 0.40 a	18.53 ± 0.76 a
	Color index	−10.71 ± 1.05 b	−10.67 ± 1.17 a	−12.53 ± 0.81 a
	Browning index	33.65 ± 1.86	48.58 ± 2.41	45.94 ± 2.66
*S. cypria*	Hue angle (°)	100.48 ± 0.81 a	95.18 ± 0.98 b	99.68 ± 0.53 a
	Chroma value	9.26 ± 0.21 b	8.77 ± 0.32 b	11.02 ± 0.57 a
	Color index	−2.60 ± 0.20 b	−1.35 ± 0.23 a	−2.69 ± 0.20 b
	Browning index	11.61 ± 0.53 b	13.00 ± 0.78 b	16.08 ± 0.99 a

Values represent the mean ± standard error (n = 5). Different Latin letters in each row indicate significant differences (*p* < 0.05).

**Table 2:** Effect of drying conditions (sun, shade, and oven) on the yield and chemical composition (%) of essential oils of *T. capitatus* plants (>0.05%).

Compound	RI	Sun Drying	Shade Drying	Oven Drying	Identification Method
α-thujene	926	1.37 ± 0.03	1.45 ± 0.09	1.28 ± 0.02	1, 2
α-pinene	933	1.26 ± 0.03	1.22 ± 0.12	1.39 ± 0.06	1, 2, 3
Camphene	948	1.35 ± 0.01	1.05 ± 0.14	1.40 ± 0.04	1, 2
1-octen-3-ol	976	0.71 ± 0.05	0.69 ± 0.08	0.85 ± 0.01	1, 2
β-myrcene	989	1.61 ± 0.06	1.89 ± 0.15	1.71 ± 0.05	1, 2, 3
α-phellandrene	1004	0.32 ± 0.01 b	0.44 ± 0.04 a	0.35 ± 0.01 ab	1, 2
α-terpinene	1017	1.41 ± 0.03 b	1.81 ± 0.14 a	1.26 ± 0.04 b	1, 2
**p-cymene**	1024	**9.04 ± 0.03 a**	**9.94 ± 1.17 a**	**7.82 ± 0.19 b**	1, 2
D-limonene	1028	1.13 ± 0.03	1.06 ± 0.12	1.13 ± 0.02	1, 2, 3
Eucalyptol	1031	0.06 ± 0.00 a	0.01 ± 0.01 b	0.04 ± 0.01 ab	1, 2, 3
**γ-terpinene**	1036	**6.71 ± 0.03 b**	**8.47 ± 0.43 a**	**5.55 ± 0.28 b**	1, 2, 3
*cis*-sabinene hydrate	1058	0.48 ± 0.01 a	0.39 ± 0.01 b	0.46 ± 0.03 ab	1, 2
α-terpinolene	1067	0.51 ± 0.01	0.50 ± 0.05	0.52 ± 0.01	1, 2
Linalool	1100	0.52 ± 0.03	0.75 ± 0.03	0.62 ± 0.08	1, 2
**Borneol**	**1166**	**4.83 ± 0.00 a**	**2.61 ± 0.33 b**	**4.75 ± 0.06 a**	1, 2
Terpinen-4-ol	1178	0.85 ± 0.05	0.86 ± 0.07	0.93 ± 0.02	1, 2
p-cymen-8-ol	1186	0.04 ± 0.00	0.04 ± 0.00	0.05 ± 0.01	1, 2
α-terpineol	1191	0.18 ± 0.02	0.18 ± 0.01	0.27 ± 0.03	1, 2
Thymol methyl ether	1239	0.80 ± 0.14	0.26 ± 0.11	0.64 ± 0.29	1, 2
Neral	1242	0.06 ± 0.02	0.07 ± 0.01	0.09 ± 0.02	1, 2
Carvacrol methyl ether	1251	0.27 ± 0.05	0.08 ± 0.01	0.23 ± 0.07	1, 2
Geranial	1271	0.09 ± 0.02	0.11 ± 0.01	0.12 ± 0.00	1, 2
**Thymol**	1290	**27.47 ± 0.84**	**29.86 ± 0.29**	**27.90 ± 0.72**	1, 2
**Carvacrol**	1300	**37.44 ± 0.97 a**	**34.56 ± 1.07 b**	**38.86 ± 0.02 a**	1, 2, 3
β-caryophyllene	1425	0.93 ± 0.05	1.00 ± 0.12	1.03 ± 0.06	1, 2, 3
Bicyclogermacrene	1502	0.03 ± 0.01 b	0.09 ± 0.00 a	0.05 ± 0.01 b	1, 2
Caryophyllene oxide	1587	0.17 ± 0.01 b	0.12 ± 0.01 b	0.25 ± 0.02 a	1, 2
Total identified		99.63 ± 0.00	99.51 ± 0.08	99.53 ± 0.06	
Grouped components					
Monoterpene hydrocarbons		24.67 ± 0.21	27.72 ± 2.39	22.34 ± 0.36	
Oxygenated monoterpenes		73.59 ± 0.18	70.27 ± 2.69	75.46 ± 0.30	
Sesquiterpene hydrocarbons		0.97 ± 0.06	1.08 ± 0.12	1.08 ± 0.05	
Oxygenated sesquiterpenes		0.17 ± 0.01 b	0.12 ± 0.01 b	0.25 ± 0.02 a	
Others		0.71 ± 0.05	0.69 ± 0.08	0.85 ± 0.01	
**EO yield**		**1.90 ± 0.20**	**1.92 ± 0.37**	**1.75 ± 0.25**	

Values are expressed as means ± standard error (n = 3). Values in rows followed by different letters are significantly different (*p* < 0.05). RI = calculated retention indices using an n-alkane standard solution, C_8_–C_20_ in ZB5 column. Identification method: 1, retention index; 2, mass spectrum; and 3, authentic compound.

**Table 3:** Effect of drying conditions (sun, shade, and oven) on the yield and chemical composition (%) of essential oils of *M. piperita* plants (>0.05%).

Compound	RI	Sun Drying	Shade Drying	Oven Drying	Identification Method
α-pinene	933	0.50 ± 0.01b	0.56 ± 0.02a	0.49 ± 0.01b	1, 2, 3
Sabinene	973	0.44 ± 0.01	0.48 ± 0.02	0.44 ± 0.01	1, 2, 3
β-pinene	977	0.84 ± 0.02 ab	0.91 ± 0.02 a	0.82 ± 0.01 b	1, 2
β-myrcene	989	0.15 ± 0.00	0.16 ± 0.01	0.14 ± 0.00	1, 2, 3
3-octanol	995	0.06 ± 0.01 b	0.07 ± 0.00 a	0.05 ± 0.00 b	1, 2
α-terpinene	1017	0.09 ± 0.00	0.08 ± 0.01	0.08 ± 0.00	1, 2
p-cymene	1024	0.07 ± 0.03	0.04 ± 0.00	0.03 ± 0.00	1, 2
D-limonene	1028	2.12 ± 0.05	2.34 ± 0.12	2.18 ± 0.05	1, 2, 3
**Eucalyptol**	**1031**	**8.20 ± 0.17**	**7.46 ± 0.29**	**7.57 ± 0.07**	1, 2, 3
*cis*-ocimene	1036	0.08 ± 0.00	0.09 ± 0.01	0.10 ± 0.01	1, 2
γ-terpinene	1058	0.21 ± 0.01	0.18 ± 0.02	0.18 ± 0.00	1, 2, 3
*cis*-sabinene hydrate	1067	0.77 ± 0.00	0.84 ± 0.06	0.74 ± 0.03	1, 2
Linalool	1100	0.13 ± 0.01	0.12 ± 0.02	0.13 ± 0.01	1, 2
Methylbutyl 2-methylbutyrate 2	1105	0.10 ± 0.00	0.10 ± 0.00	0.10 ± 0.00	1, 2
**Menthone**	**1153**	**3.30 ± 0.04 ab**	**3.78 ± 0.16 a**	**2.80 ± 0.19 b**	1, 2
***iso*** **-menthone**	**1164**	**16.25 ± 0.02 b**	**18.69 ± 0.85 a**	**16.97 ± 0.31 ab**	1, 2
**Menthol**	**1175**	**41.93 ± 0.32 b**	**43.26 ± 0.45 ab**	**44.45 ± 0.14 a**	1, 2
Terpinene-4-ol	1178	1.37 ± 0.01	1.21 ± 0.11	1.27 ± 0.08	1, 2
*iso*-menthol	1186	0.88 ± 0.01 b	0.86 ± 0.02 b	1.05 ± 0.03 a	1, 2
α-terpineol	1191	0.42 ± 0.06	0.34 ± 0.05	0.48 ± 0.11	1, 2
Pulegone	1240	1.65 ± 0.06	1.50 ± 0.09	1.68 ± 0.11	1, 2
Piperitone	1252	0.25 ± 0.01	0.23 ± 0.02	0.25 ± 0.01	1, 2
*neo*-menthyl acetate	1278	0.87 ± 0.02 a	0.63 ± 0.05 b	0.70 ± 0.04 b	1, 2
**menthyl acetate**	**1296**	**16.95 ± 0.25 a**	**14.00 ± 0.40 b**	**15.16 ± 0.74 ab**	1, 2
*iso*-menthyl acetate	1304	0.57 ± 0.00 a	0.42 ± 0.01 b	0.49 ± 0.05 ab	1, 2
β-bourbonene	1386	0.10 ± 0.00	0.09 ± 0.00	0.09 ± 0.02	1, 2
β-caryophyllene	1425	0.88 ± 0.05	0.84 ± 0.02	0.89 ± 0.08	1, 2, 3
*trans*-β-farnesene	1464	0.05 ± 0.00	0.05 ± 0.00	0.05 ± 0.01	1, 2
Germacrene D	1497	0.42 ± 0.03	0.47 ± 0.04	0.45 ± 0.04	1, 2
Viridiflorol	1594	0.04 ± 0.01	0.05 ± 0.02	0.05 ± 0.00	1, 2
Total Identified		99.62 ± 0.10	99.79 ± 0.01	99.82 ± 0.02	
Grouped components					
Monoterpene hydrocarbons		4.49 ± 0.13 b	4.82 ± 0.02 a	4.45 ± 0.02 b	
Oxygenated monoterpenes		93.51 ± 0.29	93.31 ± 0.06	93.70 ± 0.17	
Sesquiterpene hydrocarbons		1.43 ± 0.07	1.44 ± 0.06	1.47 ± 0.14	
Oxygenated sesquiterpenes		0.04 ± 0.01	0.05 ± 0.02	0.05 ± 0.00	
Others		0.03 ± 0.00	0.05 ± 0.02	0.05 ± 0.00	
**EO yield**		**1.38 ± 0.03 b**	**1.59 ± 0.06 a**	**1.48 ± 0.06 ab**	

Values are expressed as means ± standard error (n = 3). Values in rows followed by different letters are significantly different (*p* < 0.05). RI = calculated retention indices using an n-alkane standard solution, C_8_–C_20_ in ZB5 column. Identification method: 1, retention index; 2, mass spectrum; and 3, authentic compound.

**Table 4:** Effect of drying conditions (sun, shade, and oven) on the yield and chemical composition (%) of essential oils of *S. cypria* plants (>0.05%).

Compound	RI	Sun Drying	Shade Drying	Oven Drying	Identification Method
α-thujene	926	1.47 ± 0.03 b	1.59 ± 0.01 a	1.41 ± 0.02 b	1, 2
**α-pinene**	**933**	**11.49 ± 0.14 ab**	**11.21 ± 0.01 b**	**11.84 ± 0.07 a**	1, 2, 3
Camphene	948	0.05 ± 0.00	0.05 ± 0.00	0.06 ± 0.00	1, 2
**Sabinene**	**973**	**11.25 ± 0.07 a**	**11.42 ± 0.00 a**	**10.17 ± 0.19 b**	1, 2, 3
**β-pinene**	**977**	**6.10 ± 0.01 c**	**6.65 ± 0.01 b**	**8.27 ± 0.08 a**	1, 2
β-myrcene	989	1.15 ± 0.00 b	1.45 ± 0.00 a	1.14 ± 0.01 b	1, 2, 3
α-phellandrene	1004	1.93 ± 0.01 c	2.48 ± 0.00 a	2.00 ± 0.01 b	1, 2
α-terpinene	1017	0.05 ± 0.01	0.04 ± 0.00	0.05 ± 0.00	1, 2
p-cymene	1024	1.85 ± 0.06 a	1.72 ± 0.01 a	1.40 ± 0.02 b	1, 2
**β-phellandrene**	**1029**	**33.88 ± 0.48 b**	**37.72 ± 0.01 a**	**33.81 ± 0.22 b**	1, 2
γ-terpinene	1036	0.19 ± 0.06	0.10 ± 0.01	0.13 ± 0.00	1, 2, 3
*cis*-sabinene hydrate	1058	0.28 ± 0.01	0.25 ± 0.02	0.24 ± 0.00	1, 2
Terpinolene	1089	0.03 ± 0.00	0.04 ± 0.00	0.04 ± 0.00	1, 2
Linalool	1100	0.08 ± 0.02 b	0.16 ± 0.01 a	0.05 ± 0.01 b	1, 2
α-campholenal	1127	0.06 ± 0.01	0.05 ± 0.00	0.07 ± 0.01	1, 2
*trans*-pinocarveol	1139	0.05 ± 0.01	0.05 ± 0.01	0.05 ± 0.00	1, 2
Pinocarvone	1163	0.08 ± 0.01	0.09 ± 0.00	0.09 ± 0.01	1, 2
Terpinen-4-ol	1178	0.27 ± 0.01 a	0.22 ± 0.01 b	0.21 ± 0.00 b	1, 2
Cryptone	1187	2.13 ± 0.04 a	2.15 ± 0.02 a	1.16 ± 0.02 b	1, 2
α-terpineol	1191	1.02 ± 0.03 a	1.01 ± 0.01 a	0.87 ± 0.01 b	1, 2
Myrtenol	1196	0.07 ± 0.01 b	0.08 ± 0.00 ab	0.10 ± 0.00 a	1, 2
Cumin aldehyde	1241	1.16 ± 0.03 a	1.10 ± 0.02 a	0.74 ± 0.01 b	1, 2
**β-caryophyllene**	**1425**	**11.92 ± 0.16 b**	**9.74 ± 0.05 c**	**13.25 ± 0.15 a**	1, 2, 3
α-humulene	1462	0.38 ± 0.01 b	0.30 ± 0.01c	0.44 ± 0.01 a	1, 2
**caryophyllene oxide**	**1587**	**11.01 ± 0.26 a**	**8.75 ± 0.08 b**	**9.66 ± 0.24 b**	1, 2
Humulene epoxide II	1610	0.20 ± 0.02 a	0.15 ± 0.00 b	0.19 ± 0.01 ab	1, 2
14-hydroxy-Z-caryophyllene	1666	1.28 ± 0.10 b	1.06 ± 0.08 b	2.33 ± 0.24 a	1, 2
Total Identified		99.56 ± 0.05	99.54 ± 0.06	99.64 ± 0.00	
Grouped components					
Monoterpene hydrocarbons		69.67 ± 0.54 b	74.66 ± 0.01 a	70.49 ± 0.59 b	
Oxygenated monoterpenes		5.12 ± 0.04 a	4.88 ± 0.04 b	3.31 ± 0.04 c	
Sesquiterpene hydrocarbons		12.30 ± 0.17 b	10.05 ± 0.05 c	13.68 ± 0.15 a	
Oxygenated sesquiterpenes		12.48 ± 0.37 a	9.95 ± 0.15 b	12.17 ± 0.48 a	
**EO yield**		**0.21 ± 0.03**	**0.26 ± 0.03**	**0.22 ± 0.07**	

Values are expressed as means ± standard error (n = 3). Values in rows followed by different letters are significantly different (*p* < 0.05). RI = calculated retention indices using an n-alkane standard solution, C_8_–C_20_ in ZB5 column. Identification method: 1, retention index; 2, mass spectrum; and 3, authentic compound.

**Table 5:** Effects of drying conditions (sun, shade, and oven) on *T. capitatus*, *M. piperita*, and *S. cypria* EOs’ antioxidant activity and total phenolic content.

Plant		Sun Drying	Shade Drying	Oven Drying	BHT/AA
*T. capitatus*	Total Phenolic Content (mg GAE/g EO)	45.92 ± 0.92 b	65.18 ± 0.36 a	63.73 ± 0.50 a	
	Total Antioxidant Capacity (mg AAE/g EO)	4.36 ± 0.03 b	5.01 ± 0.11 a	4.39 ± 0.05 b	
	DPPH IC50 (μg/mL)	0.12 ± 0.00 b	0.12 ± 0.00 b	0.15 ± 0.01 b	1.60 ± 0.23 a
	ABTS IC50 (μg/mL)	0.10 ± 0.00 c	0.17 ± 0.00 b	0.07 ± 0.00 c	19.62 ± 0.10 a
*M. piperita*	Total Phenolic Content (mg GAE/g EO)	0.73 ± 0.01	0.69 ± 0.01	0.66 ± 0.00	
	Total Antioxidant Capacity (mg AAE/g EO)	4.69 ± 0.05	4.76 ± 0.12	4.74 ± 0.02	
	DPPH IC50 (μg/mL)	21.25 ± 0.02 a	18.39 ± 0.93 a	20.71 ± 0.78 a	1.60 ± 0.23 b
	ABTS IC50 (μg/mL)	32.43 ± 1.74 a	34.37 ± 1.39 a	19.39 ± 2.36 b	19.62 ± 0.10 b
*S. cypria*	Total Phenolic Content (mg GAE/g EO)	3.54 ± 0.01 b	4.63 ± 0.11 a	3.22 ± 0.21 b	
	Total Antioxidant Capacity (mg AAE/g EO)	6.35 ± 0.44 a	6.84 ± 0.26 a	4.40 ± 0.02 b	
	DPPH IC50 (μg/mL)	4.91 ± 0.24 a	3.15 ± 0.27 b	6.03 ± 0.27 a	1.60 ± 0.23 c
	ABTS IC50 (μg/mL)	2.24 ± 0.01 c	3.31 ± 0.06 b	3.43 ± 0.23 b	19.62 ± 0.10 a

Values represent the mean ± standard error (n = 3). Different Latin letters in each row indicate significant differences (*p* < 0.05).

**Table 6:** Effects of drying conditions (sun, shade, and oven) on *T. capitatus*, *M. piperita*, and *S. cypria* EOs’ antibacterial activity.

Plant	Bacterium		Sun Drying	SHADE Drying	Oven Drying	Streptomycin
*T. capitatus*	*E. coli*	MIC (μg/mL)	310.00 ± 0.00 a	310.00 ± 0.00 a	310.00 ± 0.00 a	3.12 ± 0.00 b
		IC50 (μg/mL)	1184.53 ± 31.19 a	1225.80 ± 37.14 a	1184.72 ± 36.80 a	20.36 ± 1.11 b
	*S. enterica*	MIC (μg/mL)	310.00 ± 0.00 a	310.00 ± 0.00 a	310.00 ± 0.00 a	3.12 ± 0.00 b
		IC50 (μg/mL)	926.22 ± 52.35 a	661.93 ± 45.71 b	557.10 ± 19.21 b	23.39 ± 1.80 c
	*S. aureus*	MIC (μg/mL)	80.00 ± 0.00 a	80.00 ± 0.00 a	80.00 ± 0.00 a	0.78 ± 0.00 b
		IC50 (μg/mL)	926.22 ± 52.35 a	661.93 ± 45.71 b	557.10 ± 19.21 b	2.33 ± 0.11 c
	*L. monocytogenes*	MIC (μg/mL)	80.00 ± 0.00 a	80.00 ± 0.00 a	80.00 ± 0.00 a	0.39 ± 0.00 b
		IC50 (μg/mL)	1029.28 ± 20.78 a	1089.30 ± 46.12 a	548.07 ± 13.17 b	2.44 ± 0.06 c
*M. piperita*	*E. coli*	MIC (μg/mL)	2500.00 ± 0.00 a	1250.00 ± 0.00 a	1250.00 ± 0.00 a	3.12 ± 0.00 b
		IC50 (μg/mL)	3596.52 ± 79.28 a	3612.33 ± 99.46 a	2890.79 ± 213.59 b	20.36 ± 1.11 c
	*S. enterica*	MIC (μg/mL)	620.00 ± 0.00 a	620.00 ± 0.00 a	80.00 ± 0.00 b	0.78 ± 0.00 c
		IC50 (μg/mL)	3055.28 ± 388.38 b	4774.81 ± 606.17 a	4328.51 ± 317.14 ab	2.33 ± 0.11 c
	*S. aureus*	MIC (μg/mL)	620.00 ± 0.00 a	620.00 ± 0.00 a	620.00 ± 0.00 a	3.12 ± 0.00 b
		IC50 (μg/mL)	1250.63 ± 142.24 a	896.83 ± 35.82 b	730.52 ± 50.38 b	23.39 ± 1.80 c
	*L. monocytogenes*	MIC (μg/mL)	160.00 ± 0.00 a	160.00 ± 0.00 a	80.00 ± 0.00 a	0.39 ± 0.00 b
		IC50 (μg/mL)	2654.73 ± 168.70 a	3072.73 ± 138.57 a	3022.03 ± 177.26 a	2.44 ± 0.06 b
*S. cypria*	*E. coli*	MIC (μg/mL)	620.00 ± 0.00 a	620.00 ± 0.00 a	620.00 ± 0.00 a	3.12 ± 0.00 b
		IC50 (μg/mL)	3287.15 ± 88.03 a	3179.77 ± 78.65 a	1616.97 ± 30.88 b	20.36 ± 1.11 c
	*S. enterica*	MIC (μg/mL)	1250.00 ± 0.00 a	1250.00 ± 0.00 a	620.00 ± 0.00 b	3.12 ± 0.00 c
		IC50 (μg/mL)	1963.23 ± 321.94 a	1493.37 ± 103.57 ab	1184.49 ± 23.68 b	23.39 ± 1.80 c
	*S. aureus*	MIC (μg/mL)	160.00 ± 0.00 a	80.00 ± 0.00 a	80.00 ± 0.00 a	0.78 ± 0.00 b
		IC50 (μg/mL)	2179.05 ± 183.21 a	1875.62 ± 569.78 a	1978.48 ± 397.66 a	2.33 ± 0.11 b
	*L. monocytogenes*	MIC (μg/mL)	620.00 ± 0.00 a	160.00 ± 0.00 a	160.00 ± 0.00 a	0.39 ± 0.00 b
		IC50 (μg/mL)	1601.68 ± 42.54 a	1784.23 ± 106.50 a	1743.61 ± 82.61 a	2.44 ± 0.06 b

Values represent the mean ± standard error (n = 3). Different Latin letters in each row indicate significant differences (*p* < 0.05).

## Data Availability

The authors declare data availability only upon request.
